# Pyroligneous acid as a multifunctional biostimulant enhances microalgal growth and soil beneficial metabolites for sustainable agriculture

**DOI:** 10.1007/s11274-025-04514-4

**Published:** 2025-08-09

**Authors:** Sudharsanam Abinandan, Praveen Kuppan, Kadiyala Venkateswarlu, Kannappar Mukunthan, Mallavarapu Megharaj

**Affiliations:** 1 Global Centre for Environmental Remediation (GCER), School of Environmental and Life Sciences, College of Engineering, Science and Environment, University of Newcastle, ATC Building, University Drive, Callaghan, NSW 2308 Australia; 2 Cooperative Research Centre for Contamination Assessment and Remediation of the Environment (crcCARE), University of Newcastle, ATC Building, University Drive, Callaghan, NSW 2308 Australia; 3 Formerly Department of Microbiology, Sri Krishnadevaraya University, Anantapuramu, 515003 India; 4 BioCarbon Proprietary Limited, Cromer, NSW 2099 Australia

**Keywords:** Wood vinegar, Biostimulant, Indole-3-acetic acid, Extracellular polymeric substances, Dehydrogenase activity, Soil health improvement

## Abstract

Microalgae are recognized as eco-functional inoculants that improve soil quality and stimulate plant productivity. This study explored the potential of pyroligneous acid (PA), a low-cost byproduct of biomass pyrolysis, as a biostimulant enhancing microalgal growth and metabolite production. We investigated the effects of PA on growth, and synthesis of indole-3-acetic acid (IAA) and extracellular polymeric substances (EPS) in two microalgal strains, *Desmodesmus* sp. MAS1 and *Chlorella* sp., when cultivated with PA in presence or absence of light. Preliminary studies showed that 0.01% (v/v) of PA enhanced growth of the microalgae, and its increased concentrations were inhibitory. Growth rate of *Chlorella* sp. with PA was 0.444 d^‒1^ in a photobioreactor compared to 0.465 d^‒1^ without PA, while the corresponding values for strain MAS1 were 0.372 d^‒1^ and 0.334 d^‒1^, respectively. Biomass production for *Chlorella* sp. and strain MAS1 when grown in presence of PA was 0.80 and 1.54 g L^‒1^, respectively, while the biomass of cultures grown in the absence of PA was 1.13 and 1.20 g L^‒1^, respectively. Chlorophyll content increased by 27% (*P* < 0.05) in *Chlorella* sp. and 17% (*P* < 0.05) in strain MAS1 in presence of PA. For *Chlorella* sp., IAA production in presence and absence of PA was 4.40 and 2.40 µg g^‒1^, respectively, while it was 6.0 and 2.0 µg g^‒1^, respectively, for strain MAS1. EPS yield increased by 2.0 mg g^‒1^ with PA in *Chlorella* sp., and the increase was 8.0 mg g^‒1^ in case of strain MAS1. *Chlorella* sp. exhibited higher chlorophyll content, enhanced IAA and EPS production but there was no increase in biomass. This suggests that PA can improve specific metabolic activities in both the microalgal strains, possibly contributing to soil health by promoting soil structure through increased EPS and IAA yield. Subsequent soil-based microcosm experiments confirmed that microalgae together with PA significantly increased soil EPS, IAA, dehydrogenase activity (DHA), and chlorophyll *a* content. The increase in DHA was 2.4-fold in soils that received PA and strain MAS1, while EPS production was 1.6 times higher. Chlorophyll *a* content was maximum in PA-amended soils inoculated with *Chlorella* sp., reaching a significant 6.5-fold increase that remained thereafter. These results highlight the dual role of PA in stimulating microalgal metabolic activity and enhancing soil microbial function, positioning it as a multifunctional biostimulant for sustainable agriculture.

**Supplementary Information:**

The online version contains supplementary material available at 10.1007/s11274-025-04514-4.

## Introduction

Microalgae are ecologically important microorganisms that play a vital role in maintaining soil structure, stability, and fertility. Through photosynthesis, they fix atmospheric CO₂ into organic carbon, improving microbial community dynamics, soil aggregation, and nutrient cycling (Jassey et al. 2022; Maier et al. 2018; Abinandan et al. 2019; Cao et al. 2023). Their biomass contains a wide range of biomolecules such as soluble amino acids, bio-mineral conjugates, polysaccharides, and phytohormones (Roque et al. 2023; Jose et al. 2024) which can stimulate plant growth and enhance tolerance to environmental stresses (Ramakrishnan et al. 2010, 2023; Rupawalla et al. 2021, 2022). Additionally, microalgae interact symbiotically with soil bacteria and fungi, thereby supporting a robust and diverse soil microbiome (Rossi and Dephilippis 2015; Chamizo et al. 2018). They also produce biologically active compounds like enzymes and ions that influence soil communities, including plants (Renuka et al. 2018). Thus, the nutrient use efficiency, plant growth, and soil microbial health positioned microalgae as promising biofertilizers and biostimulants in sustainable agriculture (Gonçalves et al. 2023). Microalgal biomass or its extracts have been linked to enhanced crop yield, stress tolerance, and reduced dependency on chemical fertilizers (Song et al. 2022; Gonçalves et al. 2023). Their integration into modern farming practices offers avenues for climate-smart agriculture, especially in low-input systems where conventional fertilizers are either unsustainable or cost-prohibitive (Miranda et al. 2024). The microalgal market initially valued at ~ 1000 million US$ in 2018, was projected to reach double by 2023 with an increase in compound annual growth rate (CAGR) of 15.20% by 2029 (Ramakrishnan et al. 2023; Miranda et al. 2024). From an economic standpoint, the scalability of microalgal production using low-cost feedstocks such as wastewater has further elevated interest in their agricultural applications (Chabili et al. 2024). Biomass generated from such dual-use systems can be valorized into biofertilizers, biostimulants, or biopesticides, promoting circular nutrient loops while lowering the environmental footprint of farming (Merlo et al. 2021; Çakirsoy et al. 2022). Despite their potential, commercialization of microalgae-based soil amendments remains limited due to challenges in cost-effectiveness, metabolite yield inconsistency, and lack of multifunctional stimulants capable of supporting both microalgal growth and soil health benefits.

Biostimulants are a diverse group of substances (Zulfiqar et al. 2020; Corsi et al. 2022) and microorganisms that, when applied in small quantities, can significantly enhance plant growth and productivity by stimulating natural physiological processes (Du Jardin 2015; Zulfiqar et al. 2020; Corsi et al. 2022; Mandal et al. 2023). They are the compounds or organisms that improve nutrient uptake, promote stress tolerance, and enhance crop quality regardless of their nutrient content (Van Oosten et al. 2017). The scientific community developed various formulations of biostimulants tailored for different crops and environmental conditions (Parađiković et al. 2019). Their integration into modern agronomic practices represents a promising strategy for achieving sustainable agriculture while mitigating the environmental impacts of conventional agrochemicals (Yakhin et al. 2017).

Pyroligneous acid (PA), also known as wood vinegar, is a byproduct obtained from the pyrolysis of lignocellulose waste or plant biomass to produce biochar (Mathew and Zakaria 2015; Akley 2023). PA, produced during biomass pyrolysis, can enhance nutrient availability, modulate bacterial communities, and improve soil properties (Cândido et al. 2023). Its chemical composition and biological effects on crops suggest potential as a biopesticide (de Souza Araújo et al. 2018; Urrutia et al. 2022). When applied at appropriate rates and concentrations, it can be used to improve crop productivity (Cândido et al. 2023; Zhu et al. 2021). For example, field experiments with lettuce, cabbage, and cucumber revealed enhanced productivity when treated with a 500-fold dilution of PA every 10 days during the vegetative growth stage (Jun et al. 2006). Further, using a 250-fold diluted PA alongside a fertilizer at half the recommended concentration resulted in a 190% increase in the number of tomato fruits and a 189% increase in their weight, compared to using the fertilizer alone (Benzon and Lee 2016). Further studies have also indicated that the application of PA also improve nitrogen fixation through action of enhancing soil nitrogen activities (Lee et al. 2021; Seok and Park 2024). However, at increasing concentrations, it can also act as a weedicide without harming any biota (Hao et al. 2021; Sivaram et al. 2022a; Iacomino et al. 2024). These findings reiterate that PA is an effective biostimulant and tested mostly as foliar spray, seed priming agent to enhance plant growth (Fedeli et al. 2022) besides promoting soil parameters and microbial diversity (Kumar et al. 2025).

The application of PA as a biostimulant in microalgal systems has not been systematically explored so far. The effects of PA on microalgal growth, metabolite release, and its potential for soil health enhancement under different nutrient conditions (autotrophy, mixotrophy, and heterotrophy) remain unknown. This is especially critical if the growing need for cost-effective and dual-benefit biostimulants that contribute to soil fertility and crop sustainability is considered. To address this knowledge gap, we used PA as the novel biostimulant for assessing its impact on microalgal growth and stimulation of soil-beneficial metabolites such as indole-3-acetic acid (IAA) and extracellular polymeric substances (EPS) by involving two microalgal strains, *Chlorella* sp*.* and *Desmodesmus* sp. MAS1. *Chlorella* sp. is well known for its autotrophic potential (Adesanya et al. 2014; Couto et al. 2021) while *Desmodesmus* sp. MAS1 has been established as a potent soil inoculant (Shanthakumar et al. 2021; Abinandan et al. 2022). Thus, these two strains were chosen for their complementary ecological traits and relevance in agricultural bioformulations while comparing their responses to PA under dark and light growth conditions. To establish the proof-of-concept, both IAA and EPS were chosen as the critical indicators of soil biostimulation because IAA promotes root elongation and nutrient uptake, while EPS contributes to soil aggregation, moisture retention, and microbial habitat stabilization. Using an optimized PA concentration (0.01%), the strains were cultivated in photobioreactors under three growth conditions: CO₂ and light (control), CO₂ and light + PA, and CO₂ and dark + PA. Also, the PA-stimulated microalgal metabolism was validated by assessing EPS, IAA, dehydrogenase activity (DHA), and chlorophyll *a* content in a soil-based microcosm study. The attenuated-total reflectance (ATR) technique was employed to assess the biochemical composition of EPS. To our knowledge, this is the first experimental evidence on biostimulation of PA, offering a novel approach to improve both microalgal productivity and soil health for sustainable agriculture.

## Materials and methods

### Microalgal strains and maintenance

The microalgal strains, *Chlorella* sp. and *Desmodesmus* sp. MAS1, maintained axenically in the Phycology Laboratory at the Global Centre for Environmental Remediation (GCER), University of Newcastle, were used in this study. The stock cultures were grown in Bold's basal medium (BBM) under continuous illumination (60 μmol m^−2^ s^−1^) at 23 ± 1 °C on an orbital shaker set at 100 rpm (Praveen et al. 2023a, b).

### Preliminary screening for growth response

PA, obtained from Northside Industries, NSW, Australia, was supplemented to BBM to provide concentrations (%, v/v) of 0.005, 0.01, 0.05, 0.10, 0.50, 1.0, and 5. About 5 × 10^5^ cells mL^−1^ collected from exponentially growing cultures of *Chlorella* sp. or *Desmodesmus* sp. MAS1 were added to 30 mL of the above growth medium in 100 mL Erlenmeyer flasks and incubated under continuous illumination (60 μmol m^−2^ s^−1^) at 23 ± 1 °C on an orbital shaker set at 100 rpm. Growth response, determined in terms of optical density (Supplementary Information, Fig. S1) and relative fluorescence units (RFUs) of chlorophyll (Supplementary Information, Fig. S2), showed that both the microalgal strains could tolerate the addition of PA up to 0.01% into the medium.

### Experimental setup and analyses

The microalgal strains, *Chlorella* sp. and *Desmodesmus* sp. MAS1, were cultivated with the optimized PA concentration (0.01%) for 8 days in 2.0 L bubble column photobioreactors. Cultures were maintained under continuous illumination (60 μmol m⁻^2^ s⁻^1^) and aerated with filtered air containing atmospheric CO_2_ at a flow rate of 0.01 vvm. The conditions included for microalgal growth were: (i) atm.CO_2_ and light with no PA (control); (ii) atm.CO_2_, light, and 0.01% PA; and (iii) atm.CO_2_, dark, and 0.01% PA, all at constant room temperature of 23 ± 2 °C. Triplicate samples were collected every alternate day, and the data were expressed as means.

The growth response was measured in terms of culture density (OD_750 nm_) and RFUs of chlorophyll using the EnSight multimode plate reader (Perkin Elmer, USA) following the method described by Perera et al. (2022). Specific growth rate (µ) during the exponential phase were calculated using the formula:

$$Specific\, growth\, rate\, (\upmu ) = \frac{\text{ln}\left({N}_{OD}f\right)-\text{ln}({N}_{OD}i)}{{t}_{f}-{t}_{o}}$$

where, N_OD_f is the optical density at tf days, N_OD_i is the optical density at day 0, and t_0_ and t_f_ are the times that correspond to the beginning and end of the exponential phase, respectively.

Biomass dry weight was determined using the gravimetric method as described earlier by Perera et al. (2022). Changes in pH were observed using a LAQUA PC1100 pH meter (Horiba Scientific, Japan).

IAA in microalgal cells was determined following the procedure of Shanthakumar et al. (2021). A known quantity of biomass was homogenized in a tissue lyser, and the supernatant was mixed with an equal volume of freshly prepared Salkowski reagent and incubated in the dark for 15 min at room temperature. The absorbance of the resulting pink colour was measured at 530 nm using a spectrophotometer. IAA was quantified using a standard curve and the values were expressed as μg g^−1^ of biomass dry weight.

For EPS analysis, at least 100 mL of supernatant was collected after 4 and 8 days of growth by centrifuging the samples at 5,000 × *g* for 15 min. The supernatant containing the released EPS was passed through 0.45 µm cellulose acetate filter. The content of EPS was precipitated by adding three volumes of ice-cold ethanol (96%) and incubated overnight at 4 °C. The precipitated EPS was centrifuged at 10,000 × *g* for 10 min, and the pellet was washed three times with 70% ethanol, followed by centrifugation at 10,000 × *g* for 10 min. The pellets were vacuum-dried, and the EPS yield was expressed as mg g^−1^ of biomass dry wt. To identify functional groups of EPS, ATR-FTIR spectroscopy was performed using triplicate samples (Perera et al. 2022). The residues of EPS were suspended in BBM to provide a concentration of ~ 2 mg mL^−1^, and spectra were obtained using an FTIR spectrophotometer (Cary 660, Agilent Technologies, Santa Clara, CA, USA). BBM without EPS was used for background spectrum to minimize the interference of solutes and traces of dissolved gases, if any, in the medium. Characteristic spectral regions corresponding to carbohydrates (900–1200 cm⁻^1^), proteins (1244–1697 cm⁻^1^), and lipids (1744, 2850–2947 cm⁻^1^) were identified as described previously (Abinandan et al. 2021).

### Soil-based microcosm experiment for validating microalgal activities

To assess the PA-stimulated microalgal impact under soil conditions, a microcosm study was conducted using sandy loam soil collected from surface (0–15 cm) in a local pasture site (pH ~ 6.5). The soil was sieved through 2-mm mesh to remove debris and homogenized. Six treatment groups were prepared in triplicate, each containing 40 g of dry soil in sterile plastic Petri dishes: (a) soil + sterile water (control); (b) soil + PA (0.01%, v/w); (c) soil + strain MAS1 (~ 30 mg dry biomass); (d) soil + *Chlorella* sp. (~ 30 mg dry biomass); (e) soil + strain MAS1 + PA (0.01%, v/w); and (f) soil + *Chlorella* sp. + PA (0.01%, v/w). The microalgal cultures were harvested on day 8 from photobioreactors and centrifuged to concentrate the biomass before soil application. PA was added simultaneously during inoculation. Moisture was adjusted to 60% of the water-holding capacity and incubated for 20 days in a glasshouse under natural light–dark cycles (~ 25 °C day/20 °C night). At day 10 and day 20, composite soil samples were collected to assess the following parameters. Soil dehydrogenase activity (DHA) was determined using the triphenyl tetrazolium chloride (TTC) reduction method and expressed as μg triphenyl formazan (TPF) g^–1^ soil hr^–1^. IAA and EPS were also quantified from soil samples as detailed above. Chlorophyll *a* was extracted from soil using 90% acetone and measured at 663 nm (Shanthakumar et al. 2021; Abinandan et al. 2022).

### Statistical analysis

The experimental mean values (*n* = 3) were subjected to one-way analysis of variance (ANOVA), and the statistical significance (*P* ≤ 0.05) was determined following Duncan’s multiple range (DMR) test using the IBM SPSS statistical software (version 24, USA).

## Results

### Growth response of *Chlorella* sp. and *Desmodesmus* sp. MAS1 in presence of PA

**Fig. 1:**
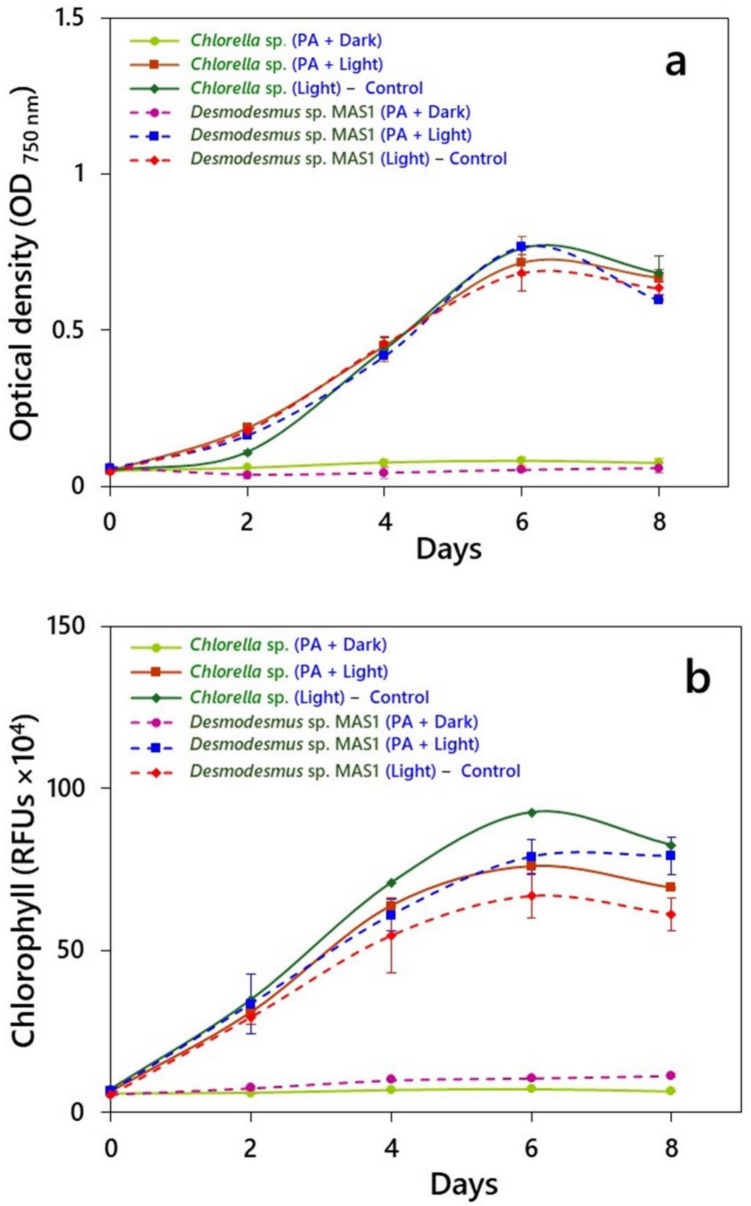
Growth, measured in terms of **a** Culture density (OD_750 nm_), and **b** Chlorophyll (relative fluorescence units, RFUs), of *Chlorella* sp*.* and *Desmodesmus* sp*.* MAS1 cultivated up to 8 days under light and dark conditions in the presence of 0.01% pyroligneous acid (PA). The data values are the means (± SD) of triplicate values

The data on growth response of *Chlorella* sp. and *Desmodesmus* sp. MAS1 in presence of 0.01% PA under light and dark conditions are presented in Fig. 1. In fact, growth patterns in cultures grown with PA were compared against positive controls (CO_2_ and light with no PA). Under the influence of PA, both the microalgal species exhibited different growth patterns, in terms of culture density and chlorophyll fluorescence depending on the cultivation mode. Both the species exhibited similar relative growth rates, determined using optical density, in light (control), with *Chlorella* sp. showing slightly higher rates when compared with *Desmodesmus* sp. MAS1 (Fig. 1a). When grown in dark, both the strains exhibited slow growth compared to other modes of cultivation, and the specific growth rates were 0.081 d^‒1^ for *Chlorella* sp. and 0.02 d^‒1^ for strain MAS1. The growth rates of *Chlorella* sp. cultivated in light with PA or without PA (control) were 0.444 d^‒1^ and 0.465 d^‒1^, respectively, while those for *Desmodesmus* sp. MAS1 the corresponding values were 0.372 d^‒1^ and 0.334 d^‒1^.

The chlorophyll content, measured in terms of RFUs, that indicate the photosynthetic efficiency, also varied significantly among different cultivation modes used in the study (Fig. 1b). Both the strains exhibited maximum chlorophyll when grown in light without PA (control), followed by their growth in presence of PA under light or dark. For *Chlorella* sp., the maximum values of RFUs were observed in control cultures, followed by those grown with PA in light or dark. In fact, the increase in fluorescence was 27% after 6 days in control cultures. Conversely, *Desmodesmus* sp. MAS1 showed maximum fluorescence after its growth with PA in light, exhibiting a 17% increase compared to the control cultures or those grown in dark at the end of 6 days. Interestingly, chlorophyll content in both the strains declined after the 6th day in control cultures, while it reached a plateau during their growth with PA in light. Overall, these results on photosynthetic efficiency suggest that both *Chlorella* sp. and *Desmodesmus* sp. MAS1 performed well in light or dark in presence of PA.

***Biomass production and changes in pH during microalgal cultivation in presence of PA*** Tables 1 and 2 summarizes the data on biomass production and pH changes when *Chlorella* sp. and *Desmodesmus* sp. MAS1 were cultivated in BBM supplemented with 0.01% PA under different growth conditions. Both the microalgal strains exhibited significant differences (*P* < 0.05) in biomass yield when cultured in presence or absence of PA in light, compared to those grown with PA in dark. In case of *Chlorella* sp., the biomass production after 8 days was maximum in control cultures, reaching approximately 1.13 g L^‒1^ followed by those grown with PA in light (0.80 g L^‒1^) and dark (0.15 g L^‒1^) (Table 1). However, the biomass production was maximum when *Desmodesmus* sp. MAS1 was cultivated with PA in light, reaching approximately 1.54 g L^‒1^ at the end of 8 days, as compared to the biomass yield in control cultures (1.20 g L^‒1^) and in cultures grown in dark with PA (0.50 g L^‒1^) (Table 1). The pH of the medium during incubation varied significantly among different cultures except for *Chlorella* sp. grown under dark conditions (Table 2). Mixotrophic growth resulted in an initial drop in pH and consequently stabilized, whereas the pH in control cultures rapidly declined initially followed by stabilization. On the other hand, the cultures of *Desmodesmus* sp. MAS1 showed significant changes (*P* < 0.05) in pH (Table 2). In cultures kept in dark, the pH remained relatively stable with a slight increase compared to the initial pH. Interestingly, pH increase was higher for *Desmodesmus* sp. MAS1 when cultured in presence of PA in light. Thus, the increase over initial pH was 17 and 12% during growth of the strain MAS1in dark and light, respectively. These findings highlight the great potential of both the microalgal strains grown with or without PA under light conditions in enhancing biomass production. The pH changes observed in the present study further indicate the specific metabolic activities within the cultures, particularly CO_2_ assimilation and photosynthesis during growth in light.

**Table 1:** Biomass yield (g dry wt. L^−1^) of microalgal strains, *Chlorella* sp. and *Desmodesmus* sp. MAS1, in the presence of pyroligneous acid (PA)

Incubation (days)	*Chlorella* sp.			*Desmodesmus* sp. MAS1		
	PA + Dark	PA + Light	Control (Light)	PA + Dark	PA + Light	Control (Light)
0	0.105 ± 0.004^ab^	0.11 ± 0.01^A^	0.12 ± 0.006^w^	0.10 ± 0.002^a^	0.11 ± 0.002^A^	0.1 ± 0.002^v^
2	0.079 ± 0.004^a^	0.15 ± 0.005^AB^	0.123 ± 0.01^w^	0.37 ± 0.001^b^	0.42 ± 0.01^B^	0.39 ± 0.08^w^
4	0.139 ± 0.005^c^	0.22 ± 0.031^B^	0.412 ± 0.03^x^	0.44 ± 0.001^c^	0.79 ± 0.04^C^	0.69 ± 0.01^x^
6	0.149 ± 0.015^c^	0.411 ± 0.033^C^	0.616 ± 0.03^y^	0.51 ± 0.013^d^	1.11 ± 0.05^D^	0.87 ± 0.07^y^
8	0.12 ± 0.0036^bc^	0.812 ± 0.034^D^	1.13 ± 0.08^z^	0.53 ± 0.02^d^	1.54 ± 0.095^E^	1.2 ± 0.04^z^

^*^The mean data values (± SD, *n* = 3) sharing the same letter are not significantly different (*P* < 0.05) from each other according to DMR test

**Table 2:** pH changes in culture medium when *Chlorella* sp. and *Desmodesmus* sp. MAS1 were grown in the presence of PA

Incubation (days)	*Chlorella* sp.			*Desmodesmus* sp. MAS1		
	PA + Dark	PA + Light	Control (Light)	PA + Dark	PA + Light	Control (Light)
0	6.51 ± 0.21	6.41 ± 0.07	6.61 ± 0.07	6.59 ± 0.14	6.68 ± 0.05	6.64 ± 0.10
2	6.46 ± 0.004	7.1 ± 0.115	7.39 ± 0.27	7.4 ± 0.225	6.39 ± 0.25	7.39 ± 0.34
4	6.29 ± 0.19	6.46 ± 0.115	7.24 ± 0.02	7.16 ± 0.11	6.6 ± 0.25	7.28 ± 0.13
6	6.67 ± 0.07	6.7 ± 0.16	6.61 ± 0.27	6.51 ± 0.065	6.98 ± 0.07	6.91 ± 0.08
8	6.56 ± 0.06	6.8 ± 0.03	6.95 ± 0.06	6.3 ± 0.15	7.04 ± 0.1	6.81 ± 0.07

### Production of IAA and EPS by microalgal cultures grown in presence of PA

**Fig. 2:**
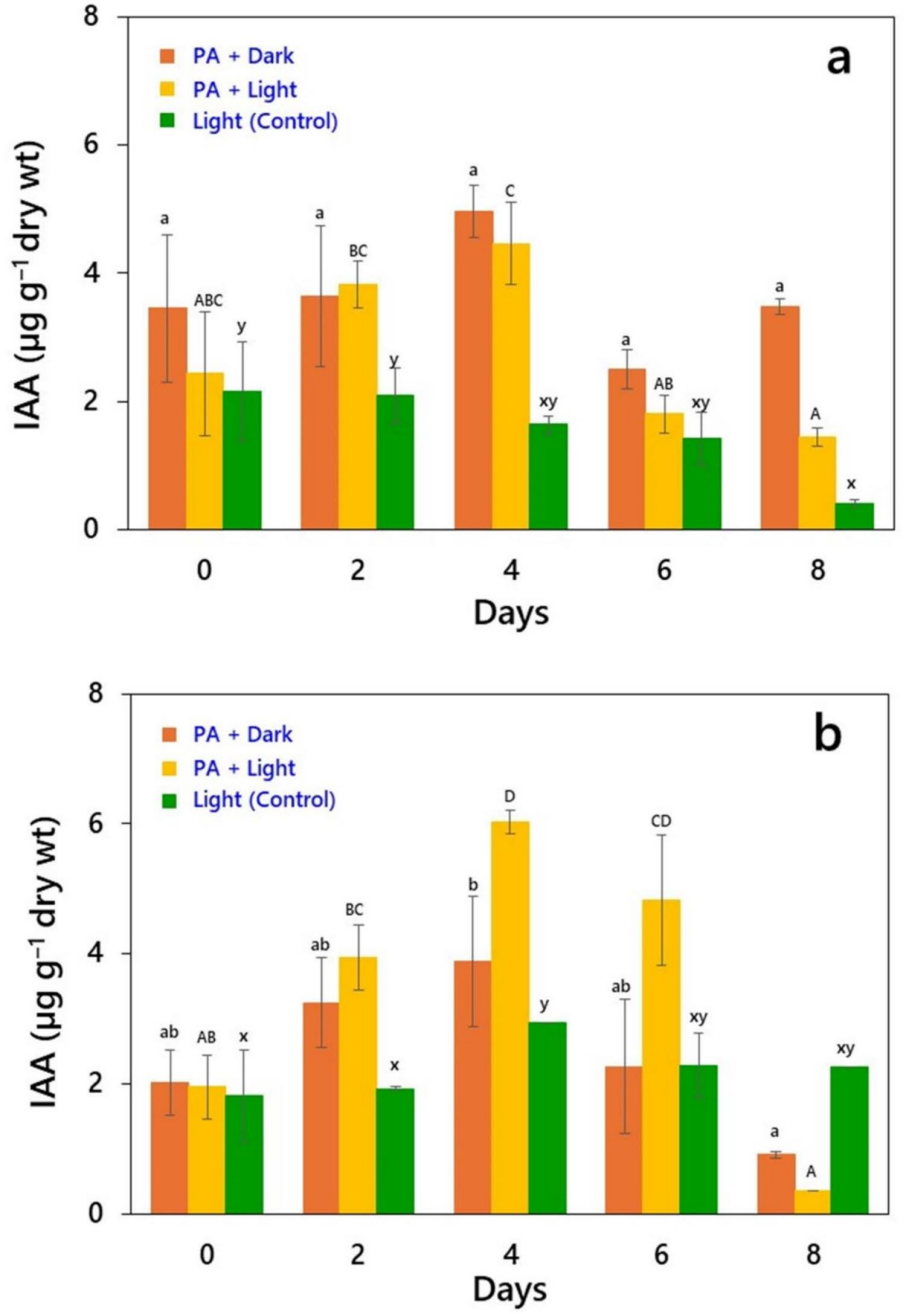
Indole-3-acetic acid (IAA) production (µg g^‒1^ biomass dry wt.) in **a** *Chlorella* sp*.*, and **b** *Desmodesmus* sp*.* MAS1 grown up to 8 days under light and dark conditions in the presence of 0.01% PA. The mean data values (± SD, *n* = 3) sharing the same letter are not significantly different (*P* < 0.05) from each other according to Duncan’s multiple range (DMR) test

The results on IAA production by *Chlorella* sp. and *Desmodesmus* sp. MAS1 grown under different modes in the presence of PA are summarized in Fig. 2. Depending on the cultivation mode, both the species exhibited significant differences (*P* < 0.05) in IAA production. *Chlorella* sp. showed higher IAA production (4.40 µg g^‒1^ of biomass dry wt) in cultures grown with PA in light when compared with the controls or those cultivated in dark in presence of PA (Fig. 2a). Thus, the increase in IAA was 83% under mixotrophic conditions (PA + light) on day 4 but subsequently decreased. However, IAA production decreased by 20% in control cultures after two days of incubation. Interestingly, the IAA synthesis was relatively higher (6 µg g^‒1^) in mixotrophic cultures of *Desmodesmus* sp. MAS1 compared to those of *Chlorella* sp. (Fig. 2b). In addition, the increase was significantly higher in mixotrophic cultures (200%), followed by 55% and 61% for those collected from dark and controls, respectively.

**Fig. 3:**
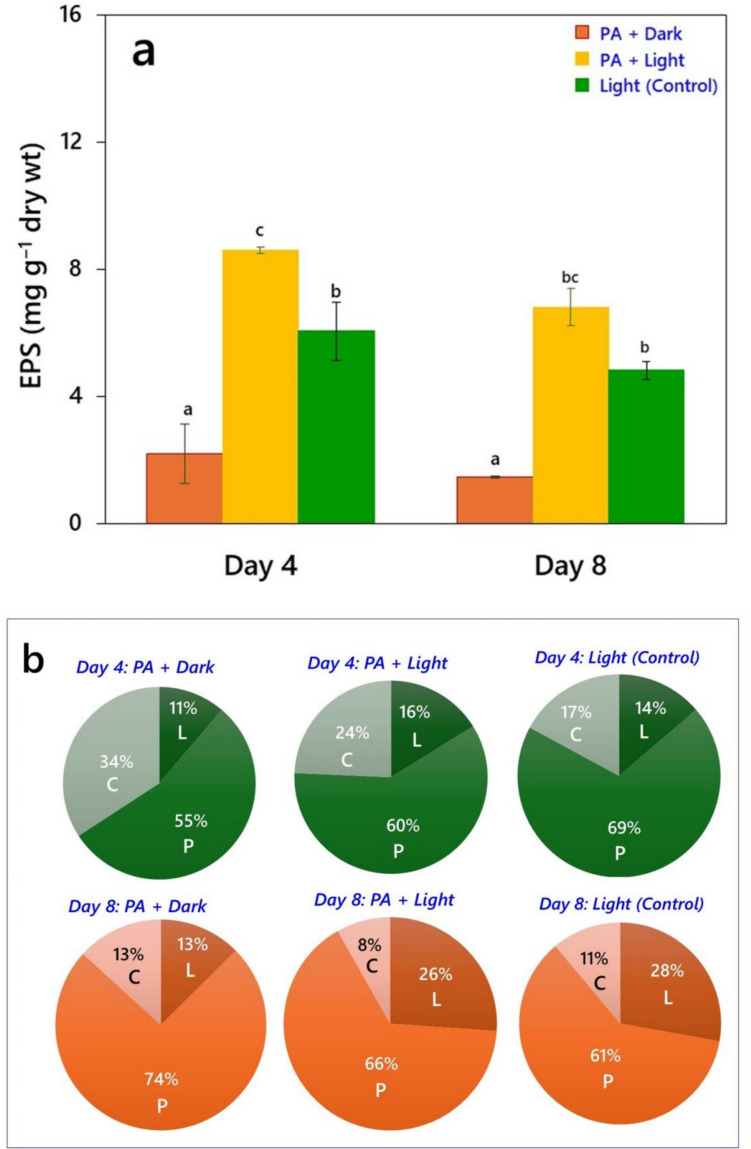
Production of extracellular polymeric substances (EPS) in *Chlorella* sp*.* when grown under light and dark conditions in the presence of 0.01% PA up to 8 days. **a** EPS yield (µg g^‒1^ biomass dry wt.), and **b** Relative biochemical composition of EPS, in terms of per cent carbohydrates (C), lipids (L), and proteins (P), measured using ATR-FTIR spectroscopy

**Fig. 4:**
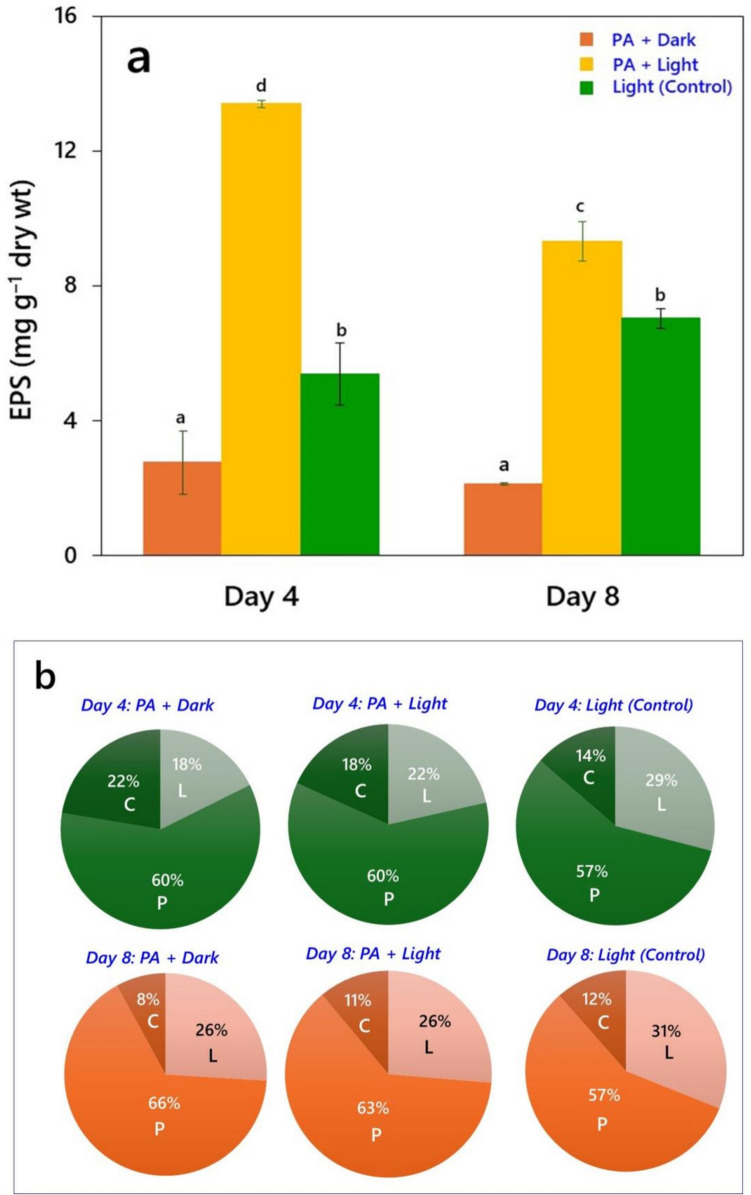
EPS production in *Desmodesmus* sp*.* MAS1 when grown under light and dark conditions in the presence of 0.01% PA up to 8 days. **a** EPS yield (µg g^‒1^ biomass dry wt.), and **b** Relative biochemical composition of EPS, in terms of per cent carbohydrates (C), lipids (L), and proteins (P), measured using ATR-FTIR spectroscopy

Based on the mode of cultivation, both the species exhibited significant differences (*P* < 0.05) in EPS production (Figs. 3 and 4). EPS production by *Chlorella* sp. was highest when grown with PA in light, reaching approximately 8 mg g^‒1^ of biomass dry wt by day 4. Growth of controls (light + no PA) resulted in production of around 6 mg of EPS g^‒1^, whereas the content of EPS produced under dark conditions was approximately 2 mg g^‒1^ (Fig. 3a). Furthermore, the biochemical composition of EPS released in dark conditions consisted of 55% proteins, 34% carbohydrates, and 11% lipids (Fig. 3b). EPS produced during mixotrophy of *Chlorella* sp. composed of 60% proteins, 24% carbohydrates, and 16% lipids, while EPS released from control cultures consisted of 69% proteins, 17% carbohydrates, and 14% lipids. Similarly, *Desmodesmus* sp. MAS1 produced maximum amounts of EPS under mixotrophic conditions (light + PA), reaching approximately 13.40 mg g^‒1^ by day 4, followed by growth in controls (5.40 mg g^‒1^) and in dark in presence of PA (2.70 mg g^‒1^) (Fig. 4a). In addition, the biochemical composition of EPS produced under heterotrophic conditions consisted of 60% proteins, 22% carbohydrates, and 18% lipids. The composition of EPS released by cultures grown mixotrophically was 60% proteins, 22% lipids, and 18% carbohydrates, whereas in controls the EPS consisted of 57% proteins, 29% lipids, and 14% carbohydrates (Fig. 4b). However, both the strains showed a 30% reduction in EPS synthesis with increased time of incubation during their growth with PA in light.

### Validation of PA-stimulated microalgal metabolism in soil microcosm

**Fig. 5:**
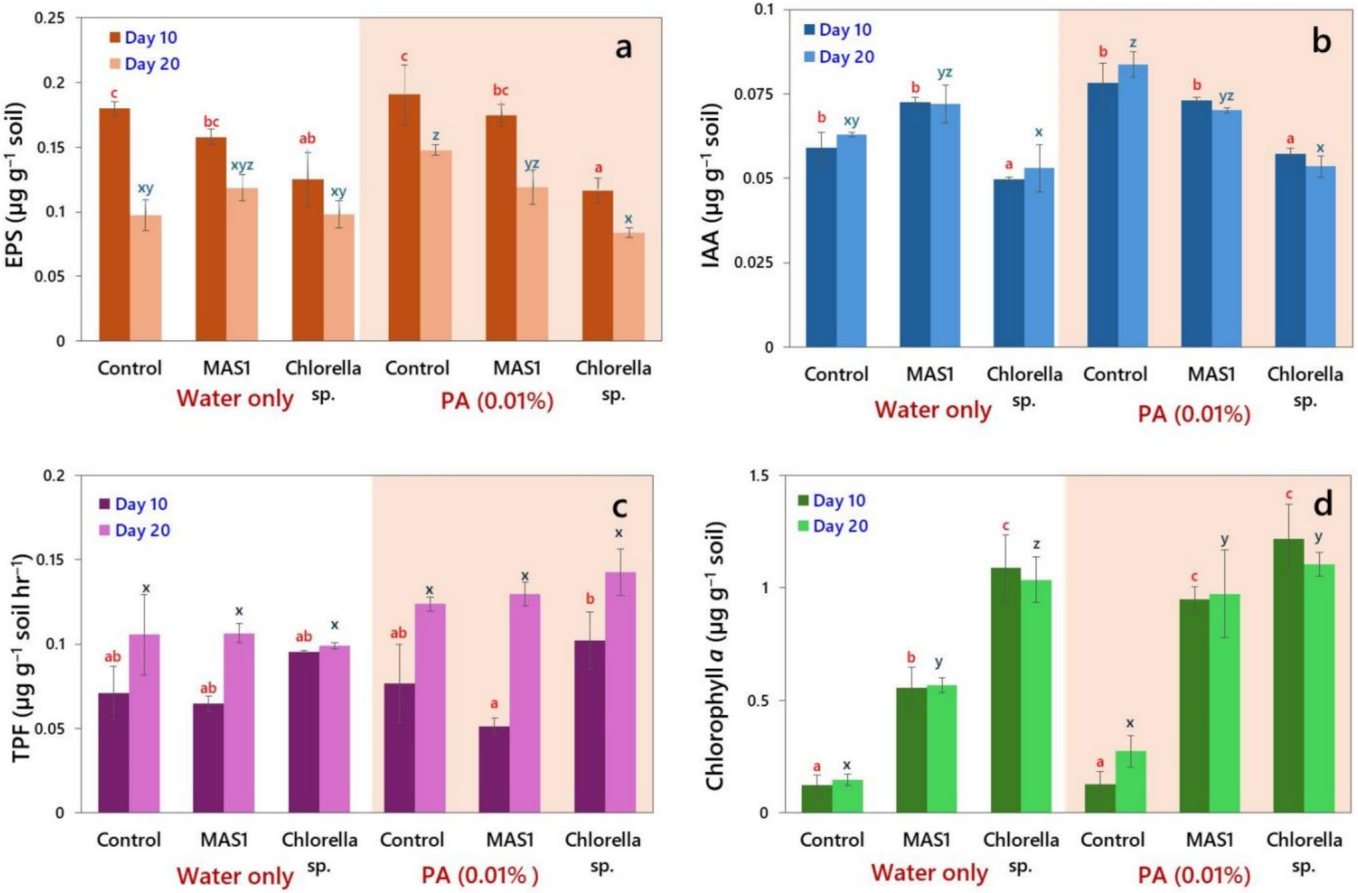
Soil-based metabolic responses following inoculation with *Desmodesmus* sp*.* MAS1 and *Chlorella* sp*.* in the absence or presence of PA (0.01%, v/w). **a** EPS production (µg g^–1^ soil); **b** IAA (µg g^–1^ soil); **c** Dehydrogenase activity (DHA, µg TPF formed g^–1^ soil hr^–1^); and **d** Chlorophyll *a* content (µg g^–1^ soil) after 10 and 20 days of incubation. The mean data values (± SD, *n* = 3) of a set of treatments sharing the same letter are not significantly different (*P* < 0.05) from each other according to DMR test

To evaluate the relevance of PA-enhanced microalgal metabolism under soil conditions, microcosm experiments were conducted using local pasture soil inoculated with *Chlorella* sp*.* or strain MAS1, with or without 0.01% PA. Results related to all four bioindicators, viz., EPS, IAA, DHA and chlorophyll *a*, demonstrated that microalgae enhanced soil microbial activities under the influence of PA (Fig. 5). The EPS content in soil was significantly (*P* < 0.05) influenced by the addition of PA in microalgal treatments (Fig. 5a). On day 10, EPS was maximum (˃ 0.18 µg g^–1^) in soil with PA alone and controls and declined by day 20 across all groups. Interestingly, EPS levels in soil with PA remained significantly higher than those in soil treated with PA and *Chlorella* sp., suggesting that PA alone stimulated EPS in soil. On day 20, lowest EPS values were recorded in soil treated with PA and inoculated with *Chlorella* sp. (0.09 µg g^–1^), indicating a possible microbial assimilation or its reduced secretion. IAA levels increased slightly from day 10 to day 20 in most of the treatments (Fig. 5b). But IAA concentration was maximum on day 20 (0.085 µg g^–1^) in soil that received only PA. IAA enhancement in soil over time was moderate with *Desmodesmus* sp. MAS1 and PA, while it was the lowest with *Chlorella* sp*.* DHA, an extensively studied indicator of microbial health of the soil, showed strong temporal increases (*P* < 0.05) across all the treatments (Fig. 5c). Soil DHA levels were maximum on day 20 with both strain MAS1 and *Chlorella* sp. treatments. Interestingly, soils treated with PA and inoculated with strain MAS1 exhibited the highest DHA with a 2.4-fold increase relative to the controls. Similarly, soil inoculated with *Chlorella* sp. in presence of PA also showed significant increases (> 1.1-fold) compared to the uninoculated controls. Chlorophyll *a*, used as a proxy for residual microalgal activity and surface photosynthetic persistence, was maximum at day 10 (~ 1.10–1.30 µg g^–1^) in soils inoculated with *Chlorella* sp. and treated with PA or without PA, and remained at elevated levels on day 20 (Fig. 5d). Similarly, strain MAS1 exhibited a two-fold increase in chlorophyll content with soils supplied with PA compared to soils with no PA. Conversely, uninoculated soil samples that received only PA had the lowest chlorophyll content, confirming that algal colonization was the primary contributor of the pigment.

## Discussion

### Impact of PA on growth of *Chlorella* sp. and *Desmodesmus* sp. MAS1

The application of PA demonstrated a positive and significant impact on the growth performance metrics, including culture density, chlorophyll content, and biomass production, in *Chlorella* sp. and *Desmodesmus* sp. MAS1. Both the strains when grown in dark in presence of PA exhibited slower growth rates, consistent with reduced chlorophyll levels due to the degradation of chloroplast membranes during nutrient assimilation (Xiong et al. 2010). In contrast, the addition of PA under light conditions significantly improved growth rates. When grown with PA, *Chlorella* sp. exhibited no significant change in growth rate compared to the control, while there was 11% increase in *Desmodesmus* sp. MAS1, indicating strain-specific responses to PA. These results are consistent with the observations of Caporgno et al. (2020), suggesting enhanced biomass production in microalgae due to the presence of both organic and inorganic carbon sources. Chlorophyll content, indicative of photosynthetic efficiency, followed a similar trend. Chlorophyll accumulation in cultures of *Chlorella* sp. grown with PA in light was comparable to that of control cultures, reflecting stable photosynthetic activity. However, *Desmodesmus* sp. MAS1 showed a 20% increase in chlorophyll content compared to the controls, highlighting its enhanced photosynthetic efficiency. These findings align with those of Murata et al. (2021), which link increased photosynthesis to carbon source availability and efficient carbon assimilation. The pH dynamics reflected the metabolic activities induced by PA. In mixotrophic cultures grown with PA, the initial drop in pH may be due to organic carbon uptake followed by stabilization due to CO₂ assimilation during photosynthesis. This pattern is consistent with the findings of Desjardins et al. (2021). Notably, *Desmodesmus* sp. MAS1 exhibited a more pronounced pH increase under mixotrophic conditions, reflecting higher metabolic activity and efficient inorganic carbon assimilation (Xiong et al. 2010). This initial drop in pH followed by its stabilization in cultures grown in presence of PA may be due to the acidic PA components and subsequent shift in the acid–base balance toward alkalinity can be ascribed to CO₂ fixation or bicarbonate assimilation during active growth (Han et al. 2022).

Biomass production was significantly influenced by PA under light conditions, although the responses varied between the strains (Vidotti et al. 2020). *Chlorella* sp. produced maximum biomass under control conditions, indicating no significant enhancement by PA. In contrast, *Desmodesmus* sp. MAS1 achieved the highest biomass yield under mixotrophy, with PA increasing its productivity by 12% as compared to the yield in controls. Higher biomass production with organic substrate in the presence of light is linked to the upregulation of genes involved in carbon flux regulation, particularly phosphoenolpyruvate carboxylase (Cecchin et al. 2018). The synergistic effects of carbon and energy metabolism under the influence of PA in the presence of light showed that microalgae could achieve higher productivity due to the combined metabolic processes of photosynthesis and organic carbon assimilation (Pang et al. 2019).

Though not yet applied in microalgal systems, PA contains phenolics and organic acids that may function similarly by serving as accessible carbon sources and biochemical triggers for growth. Several studies have shown that biostimulant such as IAA, diethyl aminoethyl hexanoate (DA-6), and gibberellic acid enhance microalgal metabolism by triggering carbon flux, improving photosynthetic pigment synthesis, and reducing oxidative stress (Jiang et al. 2015; Liu et al. 2016; Falkowska et al. 2011). The higher biomass yield and chlorophyll content observed in *Desmodesmus* sp. MAS1 may also be attributed to its ecological adaptation, in terms of metabolic flexibility, to nutrient-variable, light-exposed habitats, and possibly to more efficient mixotrophic metabolism (Praveen et al. 2023a, b). Likewise, the observed changes in chlorophyll content and biomass when grown with PA are consistent with these known stimulatory effects and suggest a potential metabolic priming mechanism. This ability of strain MAS1 to utilize complex substrates as exists in PA may have contributed to its better performance than *Chlorella* sp. However, the trade-off between biomass and chlorophyll in *Chlorella* sp. attribute to hormetic effect. Recently, Erofeeva (2023) described that trade-off due to hormetic effect can have positive, negative or zero effect on phenotypic plasticity in species response to changing environment. For instance, Stirk and van Staden (2020) reported that the phytohormones can also elicit antagonistic effect rather the heavily reported positive effect on microalgal growth. The allelochemical compound, kaempferol, at concentrations of 3.50–7.0 mg L⁻^1^ over 4 days, exerted a hormetic effect by enhancing chlorophyll content and antioxidant defense in the cyanobacterium, *Microcystis aeruginosa*, while simultaneously suppressing cell density, indicating a trade-off between defense and reproduction. But this response contrasts with the lower concentrations used (0.50–1.0 mg L⁻^1^), which stimulated cell division instead (Li et al. 2021).

Overall, the application of PA at the optimal concentration of 0.01% effectively enhanced growth rates, chlorophyll content, and biomass production while stabilizing pH in cultures of *Chlorella* sp. and *Desmodesmus* sp. MAS1. Although the response of the two microalgal strains to the supplementation of PA in culture medium is differential, our results highlight the potential of PA as a biostimulant for improving microalgal cultivation under light conditions.

### IAA and EPS production in microalgal strains under the influence of PA

IAA, a critical phytohormone, influences plant growth and development by enhancing root elongation and nutrient uptake. In this study, the presence of PA significantly stimulated IAA production in *Chlorella* sp. and *Desmodesmus* sp. MAS1 under light conditions, with MAS1 showing the highest IAA yield (6.0 µg g^‒1^ dry weight), representing a 200% increase when compared with the growth in dark. Similarly, *Chlorella* sp. showed an 83% increase in IAA production under light with PA compared to controls, while no significant effect was observed in the absence of light. This stimulation can be attributed to the complex organic compounds present in PA, which serve as sources of carbon and enhance metabolic activities related to IAA production (Zhu et al. 2021). Cellular metabolism of microalgae varies with the culture conditions, which, in turn, can change the hormone profile as well as their concentrations. Do et al. (2020a, b) showed that endogenous IAA, indole-3-pyruvic acid (IPA), and gibberellin (GA4) levels produced by *Chlorella sorokiniana* TH01 increased from 0.93, 2.66, and 3.84 μg g^‒1^ dry wt to 1.83, 3.25, and 7.88 μg g^‒1^ dry wt when the microalga was turned from phototrophic mode (no sugar added) to mixotrophic mode with 2 g L^‒1^ glucose supplement. This indicates that the addition of an organic carbon source can significantly enhance hormone production as observed in the present study. The variability in IAA production among microalgal strains and cultivation conditions aligns with the reports available in the literature. For instance, Stirk et al. (2009) observed varying IAA levels in microalgal strains depending on growth mode and phase. Similarly, Jiraskova et al. (2009) observed higher IAA levels compared to indole-3-acetamide (IAM) in *Chlorella* strains, emphasizing the influence of organic substrates in hormone production. In the present study, PA facilitated the metabolic conditions needed for higher IAA synthesis, which could promote beneficial microalgae‒soil interactions, enhance root growth, and improve nutrient uptake in agricultural soils (Rupawalla et al. 2022; Abinandan et al. 2022). These findings highlight the potential of PA in sustainable agriculture by leveraging microalgal ability to enhance soil health through hormone production.

EPS, secreted by microalgae and other microorganisms, are high-molecular-weight compounds predominantly composed of polysaccharides, proteins, and nucleic acids. These compounds play essential roles in improving soil aggregation, enhancing water retention, and protecting soil biota from environmental stress (Iacomino et al. 2024). In our study, EPS production by *Chlorella* sp*.* and *Desmodesmus* sp*.* MAS1 was significantly influenced by cultivation mode and PA supplementation. Notably, PA-induced mixotrophic conditions led to substantially higher EPS yields, supporting previous findings by Han et al. (2022) and Vidotti et al. (2020), who reported increased EPS synthesis under combined organic and inorganic carbon sources. The shift in EPS composition, particularly with an increase in lipids, observed by them may reflect enhanced carbon storage and cellular protection under PA-induced metabolic conditions, which clearly aligns with the previous findings on mixotrophy-enhanced EPS modulation. This shift, particularly the observed increase in lipids and decrease in carbohydrates under PA treatment, likely reflects changes in carbon allocation and metabolic adaptation in response to organic substrates, an effect also noted by Manhaeghe et al. (2021). Mixotrophic conditions have been shown to enhance both carbon storage and extracellular secretion, indicating that PA may function as a carbon source and stress modulator that drives EPS biosynthesis and structural complexity. Higher protein content in EPS when grown in presence of PA + light may indicate enhanced assimilation of nitrogen or biosynthesis of extracellular enzymes and stress-response proteins, a trait often observed under moderate organic carbon supplementation (Perera et al. 2022). The production of EPS by marine microalgae such as *Tetraselmis suecica* and *Cyclotella cryptica* was optimized using different carbon sources like glycerol, glucose, and acetate (Smith et al. 2020), suggesting that the mixotrophic cultivation on glycerol resulted in higher EPS yields and altered biochemical profiles.

From an agronomic perspective, the enhanced EPS yields under the impact of PA treatment have critical implications. Also, the beneficial role of EPS contributes to soil health by enhancing soil aggregate stability and promoting the formation of soil crusts, which protect the soil surface from erosion and improve water infiltration (Shanthakumar et al. 2021). The biochemical composition of EPS, which includes proteins and polysaccharides, helps bind soil particles together, creating a stable soil structure (Abinandan et al. 2019), while its ability to retain water and nutrients fosters a favourable environment for soil biota (Idowu et al. 2023). In conclusion, the application of PA not only boosted the production of IAA and EPS in microalgae but also demonstrated significant potential for improving soil health. Enhanced IAA production facilitates root‒soil interactions and nutrient uptake, while EPS contributes to soil aggregation, water retention, and microbial stability. These findings underscore the role of PA as a biostimulant in microalgal cultivation and its broader implications for sustainable agricultural practices.

### Comparative evaluation of chemical stimulants and the novelty of PA

Chemical stimulants such as phytohormones and antioxidants have shown considerable promise in improving the productivity and biochemical composition of microalgae. For example, the use of auxins like IAA and synthetic analogs such as naphthalene acetic acid (NAA) and 2,4-dichlorophenoxyacetic acid (2,4-D) has been linked to enhanced biomass, lipid accumulation, and pigment production in *Chlamydomonas reinhardtii*, *Chlorella pyrenoidosa*, and *Scenedesmus quadricauda* (Salama et al. 2014; Liu et al. 2016; Du et al. 2017). DA-6 was shown to modulate not only biomass but also protein and lipid accumulation in *Scenedesmus obliquus* and *Chlorella ellipsoidea* (Jiang et al. 2015). Similarly, the addition of gibberellic acid significantly improved metabolite profiles and photosynthetic pigments in *Chlorella vulgaris* and *Chlamydomonas reinhardtii* (Falkowska et al. 2011). Beyond phytohormones, combinations of antioxidants and signalling compounds, such as sesamol, ascorbic acid, and ethanolamine, have also been reported to improve antioxidant capacity and lipid biosynthesis, especially in docosahexaenoic acid (DHA)-producing strains like *Schizochytrium* sp. (Ren et al. 2017; Bao et al. 2022). These studies highlight how exogenous compounds can enhance carbon assimilation, reduce oxidative stress, and stimulate specific gene pathways related to metabolite production (Correa-Aguado et al. 2021). Recent advancements have explored diverse small biomolecules and phytohormone analogs as metabolic stimulants for microalgae, including dopamine (DA), melatonin, and abscisic acid (ABA) analogs, each offering unique biochemical or physiological triggers to enhance growth and metabolite accumulation. For instance, DA, a neurotransmitter compound, was shown to synergize with salinity and high light stress in *Haematococcus lacustris*, significantly boosting astaxanthin yield by 38.60% and biomass by 7.63% at optimal conditions of 25 μM DA + 1.0 g L^–1^ NaCl (Zhao et al. 2022). Similarly, melatonin, a widely distributed antioxidant and phytohormone, has been demonstrated to improve growth, lipid production, and stress tolerance across multiple strains, functioning through scavenging reactive oxygen species (ROS) and transcriptional regulation (Zhao et al. 2024). Additionally, multifunctional small biomolecules such as jasmonates, ABA, and their synthetic mimics modulate carbon flux and stimulate key biosynthetic pathways in lipid- or pigment-rich algal species (Zhao et al. 2025).

While these compounds have shown promise in controlled laboratory setups, their cost, regulatory approval, and integration into sustainable agricultural systems remain challenging. In contrast, PA, derived as a low-cost byproduct of biochar production, offers a circular solution with dual functionality, enhancing microalgal biochemical productivity of IAA and EPS, and promoting soil health through organic enrichment and microbial modulation. Unlike DA or melatonin, PA’s multicomponent composition (organic acids, phenolics, ketones, etc.) facilitates broader metabolic activation without the need for synthetic processing or purification. The significant increase in IAA (up to 200%) and EPS (up to 13.40 mg g⁻^1^) observed in *Desmodesmus* sp. MAS1 under PA-supplemented light conditions is comparable to, and in some cases exceeds, responses seen with other stimulants. For instance, previous studies reported increased IAA that ranged between 1.80 and 3.50 µg g⁻^1^ under mixotrophic growth with glucose in *Chlorella sorokiniana* (Do et al. 2020a, b). Similarly, salicylic acid and naphthoxyacetic acid increased DHA and lipid content in marine microalgae (Liu et al. 2016; Singh et al. 2018), though they are synthetic or costlier. Although the current study focused on microalgae, it is important to consider the potential influence of co-existing bacteria in non-axenic cultures. Microalgae and bacteria frequently establish mutualistic interactions, where bacteria can enhance algal growth and metabolite secretion, including phytohormones like IAA (Gonçalves et al. 2023). Bacterial pathways of auxin production, such as tryptophan-dependent routes, may have contribute to the increased IAA levels in our cultures. Furthermore, EPS composition and yield could be shaped by synergistic microalgal–bacterial exudates. Previous studies have shown that such consortia can enhance metabolite diversity and nutrient cycling, especially under stress or mixotrophic conditions (Stirk et al. 2013; Arora and Mishra 2019).

### Soil-based evidence for microalgal–PA synergy

The incorporation microalgal strains into soil in presence of PA significantly enhanced bioindicators, including EPS, IAA, DHA, and chlorophyll *a*. The most notable improvements were observed with strain MAS1 in in presence of PA, yielding the highest levels of EPS. This observation aligns with the findings of Rossi and De Philippis (2015) and Idowu et al. (2023), emphasizing EPS as a cornerstone for soil structure and biological resilience. The elevated IAA levels observed in the present study further suggest the enhanced rhizosphere functionality, supporting root–microbe interactions that are critical for nutrient cycling. Serving as a source of carbon and metabolic enhancer, PA may have stimulated the pathways of stress response and growth promotion in microalgae. In contrast, IAA production was significantly enhanced in soil that received only PA, suggesting that PA may induce or mimic auxin-like signalling via native microbial or abiotic pathways. Also, this observation may point to competitive interactions or altered metabolic pathways when microalgae are co-introduced. This is consistent with available reports suggesting that microbial consortia modulate hormone expression differently under complex amendments (Stirk et al. 2009). The significant increase in DHA with time in all treatments underscores the microbial enrichment induced by inoculation coupled with PA. The highest DHA levels (2.4-fold over control) in soil samples treated with PA and strain MAS1 affirms the synergistic effect of this strain with PA in boosting microbial respiration. On the other hand, the enhancement observed with *Chlorella* sp. + PA suggests that PA may moderately regulate enzymatic turnover in soils. Similar results of increased DHA with supply of PA at optimum levels were reported by Cardelli et al. (2020), Lee et al. (2021), and Sivaram et al. (2022b). The data on chlorophyll *a* further confirmed that *Chlorella* sp*.* maintained higher photosynthetic pigment levels than strain MAS1, particularly in combination with PA. This suggests that *Chlorella* sp. is not only more photosynthetically active but also persistent under slightly altered redox or nutrient regimes imposed by PA. These findings align with the prior reports demonstrating robust tolerance and adaptability of microalgae in variable environments (Adesanya et al. 2014; Couto et al. 2021). Interestingly, the photosynthetic ability in strain MAS1 was doubled when inoculated in presence of PA indicating the phenotypic adaptation as observed in our previous findings (Abinandan et al. 2021, 2022; Praveen et al. 2023a). Thus, the present findings confirm that PA can function as a selective biostimulant, enhancing IAA and EPS in soils without algal inoculation, but showing variable outcomes when co-applied with microalgae. *Desmodesmus* sp. MAS1 was more effective in maintaining EPS and IAA in soil when PA was absent, while *Chlorella* sp. maintained strong photosynthetic persistence. These insights highlight the importance of tailoring microalgal–PA combinations based on target soil functions and further support the use of such formulations as multifunctional biological inputs in sustainable agriculture.

## Conclusion

This study provides the first evidence that PA, a byproduct of biomass pyrolysis, can act as a multifunctional biostimulant for microalgal cultivation. At a low optimal concentration (0.01%), PA significantly enhanced growth, chlorophyll content, and biomass yield in *Chlorella* sp*.* and *Desmodesmus* sp. MAS1, particularly under light conditions. Furthermore, PA promoted the production of IAA and EPS, both of which are vital to plant growth promotion and soil structure improvement. These findings demonstrate the potential of PA-enhanced microalgae as dual-function biofertilizers that support circular, low-input agriculture. From a practical standpoint, PA offers notable advantages for large-scale applications. It is derived from waste biomass via pyrolysis, making it both economically viable and environmentally sustainable. The low effective concentration (0.01%) observed in this study implies that even small quantities could yield significant agronomic benefits, minimizing costs related to transport and handling. Moreover, PA is already produced as a secondary product in biochar facilities, enabling easy integration into existing agricultural and microalgal production systems. However, further pilot-scale studies are needed to validate the performance of PA in large-scale photobioreactors and field conditions. Consideration of storage stability, long-term soil effects, and regulatory compliance will be essential for future commercialization. Nonetheless, these results highlight PA's promising role in developing scalable, low-cost microalgal biostimulant strategies that support both crop productivity and soil health.

## Supplementary Information

Below is the link to the electronic supplementary material.

Supplementary file1 (PDF 312 KB)

## Data Availability

Data is provided within the manuscript or supplementary information.
