# Supplementary material for: Pyroligneous acid as a multifunctional biostimulant enhances microalgal growth and soil beneficial metabolites for sustainable agriculture
Source: World J Microbiol Biotechnol. 2025 Aug 9;41(8):306. doi: 10.1007/s11274-025-04514-4 (PMC12334522; doi:10.1007/s11274-025-04514-4)
Supplement: Supplementary file 1 — Supplementary file1 (PDF 312 KB) [file 11274_2025_4514_MOESM1_ESM.pdf]

Supplementary Information

Pyroligneous acid as a multifunctional biostimulant enhances microalgal growth and soil beneficial metabolites for sustainable agriculture

Sudharsanam Abinandan[1,2] • Praveen Kuppan[1,2] • Kadiyala Venkateswarlu[3] • Kannappar Mukunthan[4] • Mallavarapu Megharaj[1,2]*

[1]Global Centre for Environmental Remediation (GCER), College of Engineering, Science and Environment, University of Newcastle, ATC Building, University Drive, Callaghan, NSW 2308, Australia

[2]Cooperative Research Centre for Contamination Assessment and Remediation of the Environment (CRC CARE), University of Newcastle, ATC Building, University Drive, Callaghan, NSW 2308, Australia

[3]Formerly Department of Microbiology, Sri Krishnadevaraya University, Anantapuramu 515003, India

[4]BioCarbon Proprietary Limited, Cromer, NSW 2099, Australia

Corresponding author:

Prof. Mallavarapu Megharaj
Global Centre for Environmental Remediation (GCER)
College of Engineering, Science and Environment
University of Newcastle
ATC Building, University Drive
Callaghan, NSW 2308, Australia
E-mail: megh.mallavarapu@newcastle.edu.au
Mobile: +61 2 49138734; orcid.org/0000-0002-6230-518X

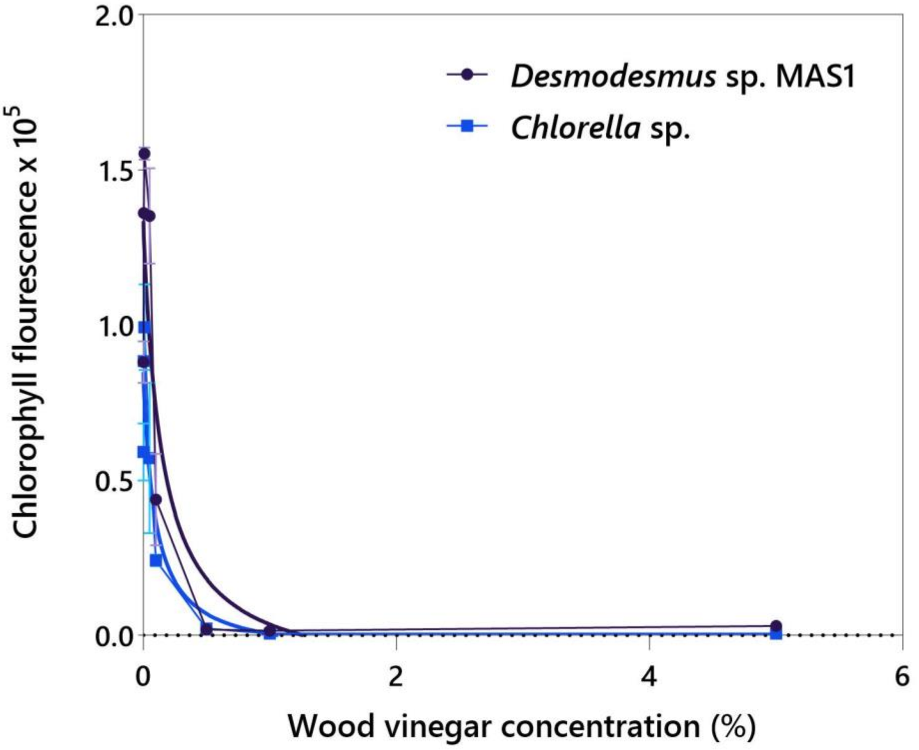

	Desmodesmus sp. MAS1	*Chlorella* sp.
Bottom	-0.1537	-0.07230
Top	1.335	0.8482
IC50	0.1455	0.09115
logIC50	-0.8372	-1.040
Span	1.488	0.9205

Fig. S1 Determination of optimal concentration of pyroligneous acid (PA) on growth (chlorophyll measured in terms of relative fluorescence units (RFUs)) of microalgal strains, *Desmodesmus* sp. MAS1 and *Chlorella* sp.

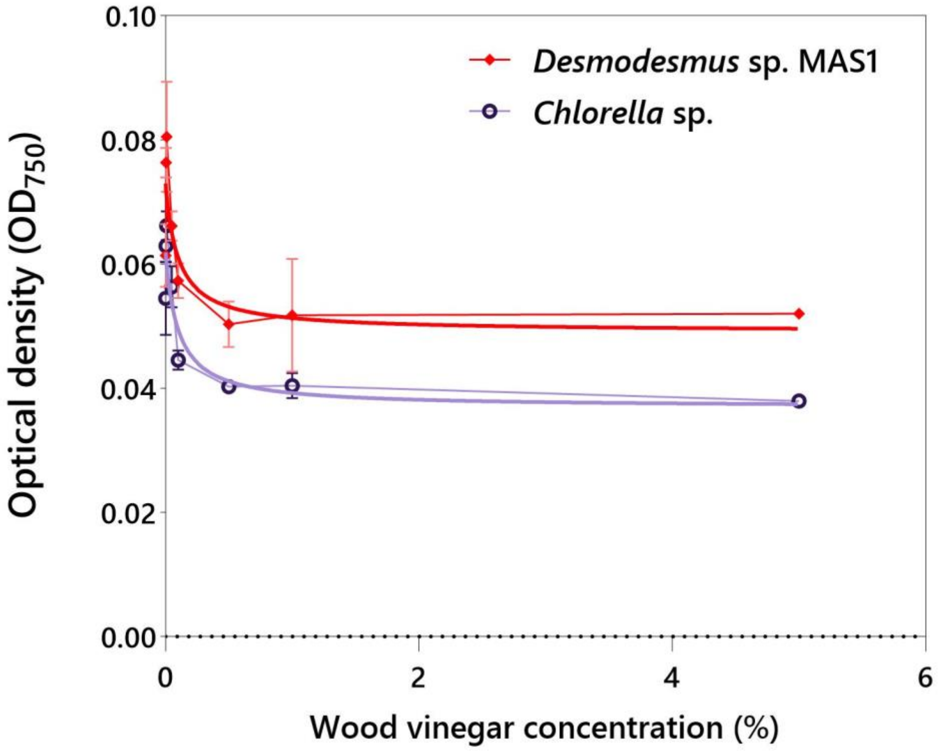

	Desmodesmus sp. MAS1	*Chlorella*sp.
Bottom	0.04914	0.03695
Top	0.07296	0.06190
IC50	0.1004	0.1006
logIC50	-0.9984	-0.9972
Span	0.02382	0.02495

Fig. S2 Determination of optimal concentration of PA on the growth (measured in terms of culture density at $OD_{750\ nm}$) of microalgal strains, *Desmodesmus* sp. MAS1 and *Chlorella* sp.
